# Application of Artificial Gastrointestinal Tract Models in Veterinary Medicine

**DOI:** 10.3390/ani15091222

**Published:** 2025-04-26

**Authors:** Sergei Konstantinovich Shebeko, Heorhii Yurievich Drobot, Andrey Georgievich Koshchaev, Svetoslav Dimitrov Todorov, Alexey Mikhailovich Ermakov

**Affiliations:** 1 Faculty of Bioengineering and Veterinary Medicine, Don State Technical University, 1, Gagarina sq., Rostov-on-Don 344000, Russia; shebeko_sk@mail.ru (S.K.S.); amermakov@ya.ru (A.M.E.); 2 Department of Biotechnology, Biochemistry and Biophysics, Kuban State Agrarian University, 13, Kalinina Street, Krasnodar 350044, Russia; kagbio@mail.ru; 3 ProBacLab, Laboratório de Microbiologia de Alimentos, Departamento de Alimentos e Nutrição Experimental, Food Research Center, Faculdade de Ciências Farmacêuticas, Universidade de São Paulo, São Paulo 05508-000, Brazil; slavi310570@abv.bg

**Keywords:** artificial gastrointestinal tract models, veterinary, microbiota studies, disease modeling, drug development, toxicology assessment, animal health, beneficial microbes

## Abstract

Artificial gastrointestinal tract models have become essential tools in veterinary medicine, providing alternatives to in vivo studies, which are labor-intensive, costly, and under certain circumstances even ethically challenging. These in vitro models facilitate the study of digestion, enable disease and host–pathogen interaction modeling, and allow for the investigation of nutrient absorption, microbiota, and pharmacokinetics. Considering the One Health concept, the application of gastrointestinal tract systems in investigations for animals can clearly reflect human health, and thus, it is pointing to the relevance of the adaptation of already existing models and the development of new models to meet the needs of veterinary and animal farming practices. This review explores and compares the various types of gastrointestinal tract models, including static and dynamic systems, and their applications across different animal species. Specific technical and methodological considerations are discussed for core animal-developed and -tested artificial systems and their integration with common ‘omics’ techniques. Dynamic models, such as RUSITEC and PolyFermS, more accurately simulate in vivo processes, including peristalsis, enzymatic activity, and microbial fermentation. The studies employing tools for ‘omics’ approaches have been conducted with more understanding analysis and comprehensive discussion and results.

## Simple Summary

The gastrointestinal tract (GIT) is responsible for nutrient absorption and overall health in animals. Its intricate structure and functions—such as mechanical digestion, enzymatic degradation, microbial fermentation, and the production of beneficial metabolites—are essential for maintaining physiological balance. However, in vivo studies of the GIT are often limited by invasive techniques, ethical concerns, species-specific variability, and high costs. As a result, GIT models have become indispensable tools for studying gastrointestinal processes. Originally developed for human research, these models have been adapted for veterinary applications. They simulate the dynamic and complex conditions of animal GIT environments, allowing researchers to examine physiological and pathological processes without the need for live animal experiments. This approach not only offers a cost-effective and ethical alternative but also reduces variability associated with studies on live animals. In veterinary research, GIT models are used to investigate nutrient digestibility, gut microbiota, and the effects of probiotics, prebiotics, antimicrobial agents, feed additives, and medications. They are also utilized in pharmacokinetics and toxicology to study drug absorption, metabolism, and the impact of feed contaminants on animal health. By advancing our understanding of gastrointestinal processes, GIT models significantly contribute to the progress of veterinary medicine while minimizing reliance on animal testing. This review examines and compares various types of gastrointestinal tract models, including static and dynamic systems, and their applications across different animal species. It highlights specific technical and methodological considerations for artificial systems designed and tested on core-animal models, as well as their integration with commonly used ‘omics’ techniques. Dynamic models, such as RUSITEC and PolyFermS, provide a more accurate simulation of in vivo processes, including peristalsis, enzymatic activity, and microbial fermentation. Studies utilizing ‘omics’ approaches have delivered deeper analytical insights, supported by comprehensive discussions and robust results.

## 1. Introduction

The gastrointestinal tract (GIT) is an essential part of animals’ bodies, where nutrient absorption and digestion take part and play essential roles in overall health [1]. Its complex structure and physiological roles, which include mechanical digestion, enzymatic degradation, microbial fermentations, and production of beneficial metabolites, are crucial for maintaining health in literally all species and have become sophisticated over long evolutionary processes [2]. Despite its importance, studying GIT in vivo presents significant challenges due to the invasive nature of the research, ethical concerns, species-specific differences, and high running costs [3]. These limitations have driven the need for alternative approaches. In this context, in vitro models offer a valuable solution for understanding GIT function, pathology, and treatment in not only veterinary medicine, but they are essential research tools in human nutrition and medical practices [4].

Artificial GIT (aGIT) models, initially developed for human research, are now being adapted and optimized for veterinary applications and investigations. These models provide controlled, reproducible environments that simulate the dynamic conditions of the GIT, including pH, enzymatic secretions, and microbial activity [5]. This allows researchers to investigate the GIT’s physiological and pathological processes without the need for live animal experimentation [6]. Moreover, in vitro GIT models offer a cost-effective and ethical solution that avoids the variability inherent in live animal studies [7].

One of the earliest aGIT models used in veterinary research was the in vitro fermentation model, which focuses on simulating microbial fermentation in the rumen of ruminants, like cattle and sheep. These static models, although useful, lack the ability to mimic dynamic processes, such as peristalsis or sequential digestion, limiting their ability to fully replicate GIT conditions [8]. In response to these limitations, dynamic GIT models, such as the Simulator of Human Intestinal Microbial Ecosystem (SHIME), have been adapted for animal research, taking into consideration the specificity of the model animal’s anatomy and physiology. SHIME, for example, allows the simulation of different sections of the digestive tract, incorporating temporal and spatial variables, making it a valuable tool for studying digestion and microbial interactions in animals [9,10].

Artificial GIT models have become common practice in veterinary medicine research. In nutrition research, they enable the study of nutrient digestibility and bioavailability in various animal species, including ruminants, monogastric animals, and avians. For instance, in vitro models are frequently used to assess the effects of different feed ingredients or additives on digestion processes [4,5]. These models are also instrumental in understanding the role of the gut microbiota, which is known to play a critical role in animal health and disease. By simulating the complex interactions between the host and its gut microbiota, aGIT models allow for the investigation of the influence of probiotics, prebiotics, antimicrobial agents, feed additives [11,12,13,14], and medications [15] on the functional processes of the animals. Beyond nutritional and microbiological research, artificial GIT models are being applied in pharmacokinetics to study the absorption and metabolism of veterinary drugs [16,17]. They are also increasingly used in toxicology, where they help assess the impact of feed contaminants and residues of veterinary drugs on animals’ GIT and health. These models represent a significant advancement in veterinary medicine, reducing the reliance on animal testing while offering precise control over experimental variables [16,18].

In this review, we will discuss the various types of artificial GIT models currently used in veterinary research, focusing on their applications in nutrition, microbiota studies, pharmacology, and toxicology. We will also address their advantages, limitations, and future potential in advancing veterinary science.

## 2. Types of Artificial Gastrointestinal Tract Models

### 2.1. Overview of Available Models

Artificial GIT models have become indispensable tools in veterinary science and can be broadly classified into static in vitro models and dynamic in vitro models, with varying levels of complexity in simulating physiological conditions, such as pH gradients, enzymatic activity, peristalsis, and microbial fermentation, and specificity related to the objective of studies and levels of simulation [19,20].

Different GIT models have been designed and explored as tools in the evaluation of functional properties of nutritional substrates and beneficial microorganisms (including probiotics), targeting applications in humans and other animals. However, it needs to be pointed out that different models described in the literature and discussed in the current manuscript are designed with specific purposes (for human or animal modeling) and later adapted for different (opposite) applications. Moreover, most of the models have been designed to explore nutritional or probiotics applications and later modified for the study of nutraceutical of pharmaceutical evaluations. Static in vitro models are typically considered for preliminary screening purposes and are simpler and designed to mimic specific segments of the GIT under control conditions, often focusing on microbial fermentation or enzymatic digestion, however, without any temporal dynamics. These systems are generally built as batch fermentation models, where substrates are incubated with microbial populations representing investigated GIT sectors with fixed time frames under controlled temperatures (specific for modeled animal) and anaerobic (or microaerophilic) conditions. While static models offer reproducibility and have reasonably been easy to use, the limitations are related to lack of the ability to replicate the dynamic processes of the digestive tract, such as the continuous passage of digesta, changing pH, or the gradual enzymatic breakdown of nutrients [21]. However, these models are particularly useful for evaluating the fermentative capacity of ruminant species, such as cattle and sheep, as well as assessing the digestibility of different feed components in monogastric animals, for instance, pigs.

Dynamic in vitro models, considered as closer to the reality of digestive systems, attempt to simulate the continuous, time-dependent processes of digestion, peristalsis, and even some specific microbial interactions that are part of the GIT environment. These models incorporate mechanisms that mimic peristalsis, sequential digestion, and variable physiological conditions across different GIT compartments [22,23,24]. Notable dynamic models include the TIM-1 (TNO Intestinal Model) and the SHIME (Simulator of the Human Intestinal Microbial Ecosystem) and have even been designed to be human GIT models; different variations have been adapted to the needs of animal research projects. Both systems use a series of interconnected compartments to simulate the flow of digesta through the digestive tract, allowing for the sequential exposure of feed or nutrients to different pH environments, enzymes, and microbial communities, while also simulating physical processes, such as transit time and nutrient absorption [25,26,27]. In addition to standard physiological and biochemical processes, these models are particularly valuable for studying the microbiota, as they allow for long-term fermentation studies and more accurately reflect specific in vivo conditions across the entire digestive system.

### 2.2. Static Versus Dynamic Models

Static in vitro models are primarily used to study isolated segments of the digestive tract, such as the stomach or the rumen, and have and still serve as preliminary screening evaluation models. In these models, specific sections of the GIT are recreated in a controlled environment, allowing the researcher to focus on a singular aspect of digestion, such as microbial fermentation or enzymatic hydrolysis of feed substrates [28]. One of the most widely used static models is the batch fermentation system, which is particularly effective in replicating the anaerobic conditions of the rumen to study the fermentation of fibrous feeds. This specific model is limited and does not represent dynamic conditions or the real variety of processes in ruminants. It lacks the ability to simulate the progressive breakdown of nutrients, fluctuations in pH, temperature regulation, and transit of digesta—factors that are essential for mimicking the natural digestion process. However, it is simple, easy to control and manipulate, and serves as a preselection screening model, where high numbers of tests can be performed for further research [29,30]. Static models are ideal for high-throughput screenings of feedstuffs and are commonly employed to investigate feed additives, probiotics, and prebiotics.

In contrast, dynamic models attempt to replicate the entire digestive process by incorporating features, such as gradual nutrient input, automated pH control systems, temperature regulation, and mechanical elements that simulate peristaltic contractions and digesta transit, thereby mimicking the temporal and spatial variability across various sections of the in vivo gastrointestinal tract. Most of the dynamic models were originally designed for the need to simulate human digestive systems; however, later, most of them were adapted to the specificity and needs of veterinary medicine and animal research practices. For instance, the SHIME model simulates the stomach, small intestine, and large intestine, each represented by distinct compartments. The SHIME system is particularly valuable in simulating both the upper and lower GIT, offering a more holistic view of digestion and microbial interactions across time [10,27]. The TIM-1 model goes a step further by mimicking the specific physiological conditions of the upper digestive tract (stomach and small intestine), including changes in pH, bile salt concentrations, and enzyme secretions [31,32]. These dynamic models, by more closely replicating in vivo digestion, are particularly useful in studying nutrient absorption, the interaction between the gut microbiome and host, and the pharmacokinetics of veterinary drugs.

Although dynamic models offer a closer approximation of real digestive processes, representing better intestinal processes, they are more technically demanding, associated with specific hardware (and even software), need to be manipulated by trained researchers, and are more expensive compared to static models [33]. Dynamic models often require sophisticated apparatuses capable of controlling and adjusting environmental conditions to simulate GIT’s ever-changing physiology [34]. Despite these challenges, dynamic models provide crucial insights into the kinetics of digestion and fermentation that static models cannot, making them particularly valuable in complex research surrounding nutrition, gut health, and microbial ecology.

### 2.3. Animal-Specific GIT Models

One of the significant developments in aGIT models has been the adaptation of these systems to replicate the digestive processes of specific animal species [35]. This species-specific approach is critical due to the vast differences in GIT anatomy and function across ruminant, monogastric, and avian species, fish and other aquatic farm animals, as well as carnivores, like cats and dogs [36]. Moreover, the specific body temperature has always been an accent in the adaptation or creation of new models.

Ruminant-specific models are particularly well developed, given the complexity of ruminant digestion, which involves multi-compartmental fermentation in the rumen, reticulum, omasum, and abomasum [37]. These models, such as the Rusitec (Rumen Simulation Technique), simulate the rumen’s anaerobic fermentation process, allowing for prolonged fermentation studies that closely replicate ruminal conditions. The inclusion of ruminal microbes in these models enables researchers to investigate the effects of dietary changes, feed additives, and antimicrobial agents on ruminal fermentation, fiber degradation, and methane production [38,39].

Monogastric models, used primarily for pigs and dogs, are simpler in structure but still require adaptations to replicate species-specific digestive physiology [40]. For example, pigs, often used as models for human digestion, have been the focus of modified SHIME and TIM-1 systems that mimic their GIT. These models account for the distinct characteristics of monogastric digestion, such as gastric acid production and enzyme activity in the small intestine. They have been employed in research on nutrient absorption, probiotic efficacy, and gut health, with a particular emphasis on piglet nutrition and early weaning [41].

Avian GIT models represent an additional challenge due to the unique structure of the avian digestive system, which includes specialized organs, like the crop, gizzard, and proventriculus [42,43,44]. Artificial GIT models for poultry and other farmed or companion birds have been developed to simulate these unique compartments and their associated microbial populations. The poultry-specific in vitro models, for instance, focus on mimicking the rapid transit time and high metabolic rate characteristics of avian species. These models have proven particularly useful in assessing the impact of dietary changes on poultry growth performance and gut health, as well as in studying the role of probiotics and antibiotics in modulating the gut microbiome [45,46,47].

Moreover, models designed for carnivorous species, like cats and dogs, are still relatively underdeveloped but are gaining attention due to the rising interest in pet nutrition and health. These models aim to replicate the shorter, more acidic digestive tracts of carnivores, which are adapted for a protein-rich diet [48,49,50]. Current research in this area focuses on simulating protein and fat digestion, gut microbiota composition, and the absorption of nutrients from commercially available pet foods [51].

### 2.4. Animal-Specific aGIT Model’s Environment

Artificial gastrointestinal tract models have become indispensable tools for replicating species-specific digestive environments under controlled laboratory conditions. These systems enable the precise investigation of digestive physiology, microbial ecology, and dietary interventions across monogastric and ruminant species.

For poultry, the CALIMERO-2 model provides a dynamic simulation of the chicken cecum at 41 °C and pH 6.6 under anaerobic, nitrogen-flushed conditions. It integrates peristaltic mixing, pH control, and a dialysis membrane to prevent metabolite accumulation while preserving physiological short-chain fatty acid (SCFA) concentrations. Fed with Standard Ileal Effluent Medium (SIEM) and its poultry-adapted variants, the model sustains native microbial consortia, such as *Bacteroidetes* and *Firmicutes*, producing in vivo-like metabolite profiles [46]. Complementing this, the Enteromix Chicken Cecum Simulator simulates the complete chicken gastrointestinal tract, from crop to cecum. It incorporates sequential enzymatic digestion phases and a time-resolved anaerobic cecal compartment maintained at 39 °C, where feed type and inoculum freshness significantly influence microbiota and SCFA output, confirming the model’s adaptability and sensitivity [45]. For canines, a semi-dynamic in vitro model replicates the gastric phase with a gradual pH reduction (from 5.4 to 1.9 over 3 h) and controlled enzyme addition (pepsin and lipase) at 39 °C. Despite a static intestinal phase, the system effectively assesses nutrient bioaccessibility and enzymatic digestibility, with supplementation studies demonstrating enhanced digestive efficiency [51].

In ruminants, the RUSITEC system was used to simulate the bovine rumen environment over a 15-day period. Key physiological conditions, such as anaerobiosis (achieved via nitrogen flushing), constant buffer inflow, and temperature maintenance at 39 °C, were employed. Despite the absence of host-related factors, like absorption and protozoal populations, the system maintained stable fermentation parameters (pH, redox potential, SCFA, and ammonia levels). Introduction of the pathogen *Clostridium perfringens* led to elevated amino acid concentrations due to proteolytic activity, although without disrupting the overall bacterial composition [39]. Camacho et al. [30] expanded this concept by developing a standardized in vitro digestion protocol using DaisyII and TE-150 fermenters. These systems simulated ruminal conditions using rumen inoculum and buffer solutions at 39 °C, with rotary agitation enhancing microbial–substrate interactions. The study found that vessel design significantly impacted digestibility metrics, underscoring the importance of equipment standardization across laboratories to improve reproducibility in simulating ruminal digestion.

The dynamic multicompartmental model [25] simulates four sequential compartments—stomach, duodenum, jejunum, and ileum—reproducing gastric emptying kinetics (t½ = 35–70 min) and ileal transit (t½ = 85–160 min) via computer-controlled peristaltic pumps. The gastric pH decreases progressively from 4.8 to 1.7 over two hours, while the duodenum maintains pH 6.5. Bile salt concentrations are tightly regulated: 10–15 mmol/L initially in the duodenum, 10 mmol/L in the jejunum, and 2–4 mmol/L in the ileum. Hollow-fiber dialysis units simulate intestinal absorption, achieving 96% glucose removal, closely reflecting in vivo uptake.

## 3. Applications for Veterinary Medicine

### 3.1. Disease Modeling and Host–Pathogen Interaction Studies

Static and dynamic artificial GIT models are widely used in medical and biological research in the field of pathology and physiology [52]. The development of GIT models has revolutionized the study of probiotics by providing controlled environments to evaluate their beneficial effects on gut health. These models simulate dynamic GIT conditions, such as microbial fermentation and enzymatic activity, enabling researchers to investigate probiotic interactions with gut microbiota, nutrient absorption, and the production of beneficial metabolites. By offering ethical, cost-effective, and reproducible alternatives to in vivo studies, GIT models play a pivotal role in advancing our understanding of probiotics and their applications in animal and human health, where their design and development was extended to meet the needs of probiotics research. They allow detailed modeling of various gastrointestinal diseases as well as individual mechanisms of their development. The ability to model infectious and non-infectious disease processes in a controlled environment allows us to study the features of pathogenesis, pathogen behavior, host–pathogen interactions, the impact on microbiocenosis, and the effectiveness of therapeutic interventions without endangering humans and animals [5]. As a rule, modeling of diseases and studying pathogenesis in conditions of artificial digestive systems is one of the elements of research devoted to studying the effectiveness of pro-, pre-, and postbiotic agents and various biologically active substances [5,53,54,55,56,57]. For instance, knowledge obtained and summarized in different studies, in particular, regarding spore-forming probiotics [58,59] or successful inhibition of the pathogen [60] could be applied in certain GIT models for more comprehensive research.

Moreover, GIT models serve as valuable tools in education, offering students hands-on opportunities to explore complex digestive processes in a controlled and ethical manner. These models simulate dynamic GIT conditions, such as microbial fermentation and enzymatic activity, enabling learners to understand physiological and pathological mechanisms without the need for live animal experiments. By integrating GIT models into teaching, educators can enhance the comprehension of topics, like nutrient absorption, gut microbiota interactions, and the effects of dietary interventions, fostering a deeper understanding of gastrointestinal health and its implications in veterinary and biomedical sciences [61].

Most studies in this field focus on the impact of pathological factors on disease development in humans, since they have been implemented based on systems simulating the human digestive tract, such as TIM-2, SHIME, and their modifications, according to the specificity of animals’ anatomy and physiology. Studies aimed at animals are much less common. Thus, Asare et al. [62] reported on results regarding the evaluation of the microbiota of chickens under healthy conditions using the PolyFermS model. During the study, the system qualitatively mimicked the chicken cecum and proved to be a useful tool for studying microbiota ex vivo. The metabolic profile remained stable over the monitored period, with acetate (53.2%), propionate (7.1%), and butyrate (14.2%) as the main SCFAs. The microbiota composition included *Firmicutes* (79.9%), *Bacteroidetes* (13.9%), and *Proteobacteria* (4.5%), closely mimicking in vivo conditions. The optimized modified *Viande Levure* medium (mVL-3) supported microbial activity, maintaining stable metabolite concentrations (174.5 ± 10.5 µmol/mL in fermentation No. 2, 142.1 ± 5.9 µmol/mL in fermentation processes). We should agree with the authors of this publication since it is obvious that installations simulating some individual sections of human and animal GIT, for example, the colon, are built on the same principles and are similar in most parameters and design. Therefore, PolyFermS and similar systems are most suitable for such studies in veterinary medicine.

PolyFermS has also been used to study *Salmonella* colonization in the swine proximal colon under the influence of certain probiotics [53]. In this study, the system qualitatively simulated the conditions of animal intestine infection, monitored pathogen colonization, and evaluated the effectiveness of the research objects without involving live pigs in the experiment. In the context of studying infectious disease pathogenesis, gastrointestinal models can also allow researchers to study pathogen–host interactions. For example, Chang and Chen [63] demonstrated that a lactobacilli mixture reduced *Campylobacter jejuni* populations in the stomach and small intestine of an aGIT model, providing insights into probiotic-based pathogen control. Card et al. [64] showed that a simulated chicken gut environment facilitated the transfer of a multidrug resistance plasmid from *Salmonella* to commensal *Escherichia coli*, highlighting the risks of horizontal gene transfer in poultry microbiomes. Mycotoxin binding studies using artificial gut models have identified optimal adsorbents. Tso et al. [65] tested seven commercial mycotoxin removers, showing that enzyme degradation reagents removed more than 50% of deoxynivalenol and zearalenone, whereas traditional adsorbents were less effective. Additionally, prebiotic interventions were tested using an in vitro fermentation system. Donalson et al. [66] observed that fructooligosaccharide supplementation increased cecal acetate and volatile fatty acid production, enhancing microbial diversity and gut health.

Moreover, TIM-2 and SHIME have been adapted to animals’ anatomy and physiology with the aim of modeling pathogen colonization and mucosal infection in various parts of the gastrointestinal tract [5]. A study using the MPigut-IVM model [67] has investigated host–pathogen interactions in piglets, particularly in relation to enterotoxigenic *Escherichia coli* (ETEC) infections under a variety of environmental conditions, such as varying pH levels and nutrient availability. The representatives from the genus *Morganella* increased 4-fold post-ETEC challenge, aligning with its association with diarrhea. IPI-2I cells exposed to ETEC-conditioned medium showed a 2.5-fold increase in MyD88 (immune signaling), a 3-fold increase in TNF-α (inflammatory cytokine), and a 1.8-fold increase in MUC1 (mucin production). Claudin-4 expression increased 2.2-fold, indicating tight junction modulation due to infection. These findings illustrate how the use of MPigut-IVM helps link microbial and host responses by quantifying gene expression changes in epithelial cells under controlled infection conditions. Higher tyramine and valerate and lower ethanol levels correlated with inflammatory responses. The simulation of weaning via 48 h feed deprivation and dietary shift altered microbial composition in MPigut-IVM: e.g., *Prevotellaceae* increased from 6.5% to 27.1%, while *Bacteroidiaceae* decreased from 24.9% to 10.3%. SCFA levels also shifted, with butyrate decreasing and propionate rising, indicating functional remodeling of microbiota post-weaning. Using another in vitro model, the researchers exposed fecal microbiota from healthy children to six toxigenic strains of *C. difficile* or their conditioned media [68]. All *C. difficile* strains demonstrated high sporulation rates (20–57%) in the presence of children’s gut microbiota, which was significantly higher than that in control media (<8%). Exposure to *C. difficile* led to a substantial decrease in microbial diversity, particularly with ribotypes 027 and 176. The abundance of butyrate-producing families, such as *Lachnospiraceae* and *Ruminococcaceae*, declined, while opportunistic pathogens, like *Escherichia* spp. and *Klebsiella* spp., increased. Usage in vitro systems can be applied to the development of new principles for targeting pathogen colonization in livestock, including the use of antimicrobial peptides, bacteriophages, and immunomodulatory feed additives. A study [56] has examined the antibacterial activity of Microcin J25 (MccJ25), a peptide antibiotic produced by *E. coli*, against *Salmonella* Newport in an in vitro continuous fermentation model (PolyFermS) simulating swine proximal colon conditions. The minimal inhibitory concentration (MIC) and minimal bactericidal concentration (MBC) of MccJ25 in LB medium were 0.03 µM and 3.71 µM, respectively. These values were significantly lower than those of reuterin (MIC = 1078.91 µM, MBC = 4315.63 µM) and rifampicin (MIC = 37.97 µM, MBC = 1215.15 µM), indicating the superior efficacy of MccJ25. The mentioned models may allow researchers to simulate the effects of gastrointestinal inflammation and other pathological conditions, such as colitis or enteritis, which are common in livestock and domestic animals. By introducing inflammatory triggers (e.g., lipopolysaccharides, cytokines) into the model, researchers can study the inflammatory cascade and its impact on gut barrier function, nutrient absorption, and microbiota composition. This approach is promising for testing novel anti-inflammatory drugs, gastroprotectors, and nutraceuticals that could mitigate GIT inflammation. As an example, research utilizing the SHIME model [69] concluded that Aronia polyphenols modulate intestinal microbial composition, induce beneficial SCFA production, and prevent inflammatory stress in endothelial cells.

Thus, there is no doubt that the pathophysiological direction in scientific research in veterinary medicine using artificial GIT models is very promising and in demand. Taking into consideration the One Health concept, the application of GIT models in investigating animals’ health can clearly reflect human health, and thus, it points to the relevance of the adaptation of already existing models and the development of new models for the needs of veterinary and animal farming practices.

### 3.2. Drug Development and Testing

Artificial GIT models are promising test systems at various stages of veterinary drug development, particularly for evaluating pharmacokinetics and pharmacodynamics without the ethical concerns of in vivo testing and significant financial costs. Currently, this area is just beginning to develop in veterinary medicine and animal farming practices. However, in human medicine, it is already a widely accepted approach, and most probably, at this stage, the adaptation of already well-established human models are the subject of research interests and applications. Numerous studies utilizing the above-mentioned GIT systems (primarily SHIME, TIM-1, TIM-2) and the SIMGI instrument, an automated gastrointestinal in vitro model designed to dynamically simulate the physiological processes of humans’ GIT [27], are described in available sources, focusing on the evaluation of the effectiveness and bioavailability of various biologically active substances of synthetic pharmaceuticals of chemical and natural origins [69,70,71,72,73,74]. These models not only allow for the accurate assessment of the effects of the studied objects on GIT parameters, processes occurring within it, and the microbiota but also enable the study of drug absorption, metabolism, and biopharmaceutical properties in a controlled environment, simulating conditions of different sections of the GIT [75]. This research utilized the TIM-1 system and showed that paracetamol absorption was faster when it was administered as a free powder than in sustained-release tablet form, and a slow passage time resulted in a delay in the absorption of paracetamol. TIM is an advanced in vitro model that simulates the dynamic conditions of the GIT and was initially designed for the simulation of the human intestinal system [25]. It has been widely adapted for veterinary practices [31,76,77] and is widely used for the evaluation of drug absorption, metabolism, and efficacy in both human and veterinary medicine. The TIM model offers a controlled environment to study the interactions between drugs and the GIT, providing valuable insights into their pharmacokinetics and pharmacodynamics, since the model replicates the physiological conditions of the GIT, including pH, temperature, peristalsis, and the presence of digestive enzymes and bile salts. The model allows for the continuous monitoring of drug release, absorption, and metabolism, providing a comprehensive understanding of drug behavior in the GIT.

The TIM model is used to evaluate the absorption and bioavailability of veterinary drugs. By simulating the GIT conditions of different animal species, the model helps in predicting the extent and rate of drug absorption. This information is crucial for determining the optimal dosage and administration route for veterinary drugs, ensuring their efficacy and safety. Moreover, the model is also employed to study drug–drug and drug–food interactions in veterinary medicine, allowing the investigation of mechanisms for how different drugs and food components affect the absorption and metabolism of veterinary drugs. This information is essential for developing guidelines on drug administration and dietary recommendations for animals [31,75].

Was suggested that such modifications of the TIM model as TinyTIM and TIM-agc can be applied to assess the performance of different drug formulations in the GIT, where by comparing the release and absorption profiles of various formulations, researchers can identify the most effective and stable formulations for veterinary drugs [31].

An artificial stomach–duodenum (ASD) in vitro model was described in study [78]. It was designed to simulate the pH-reduced canine stomach to assess poorly soluble free acid compounds. The model maintained duodenal pH control using NaOH infusion, allowing for an accurate in vitro–in vivo correlation. Five formulations with different solubilization mechanisms were tested, showing a high correlation (r^2^ = 0.987–0.989) between in vitro and in vivo areas under the curve when using simulated intestinal fluids. Raman spectroscopy identified drug gelation during dissolution, with hypromellose stabilizing the gelled state to prevent further solid conversion. So, this work highlights the ASD model’s reliability for formulation assessment and mechanistic drug performance studies.

With the development of the concept of use of pre-, pro-, or postbiotics, research has focused on the evaluation of the effectiveness of those additives and the benefits for animals’ health [53,79,80,81,82,83]. Pre-, pro-, and postbiotics are attracting researchers’ and industry attention, related to their benefits in improving general health and the yield quality of animal products [84,85,86]. In addition, pro- and postbiotics have been considered and are already effectively applied as alternatives to antibiotics, as growth promotors, and in prevention of some bacterial diseases in farming animal practices. Evaluation of the effectiveness of pro- and postbiotics include evaluations in appropriate models. For example, Marzorati et al. [54] investigated the effects of MegaDuo™, a probiotic formulation containing Bacillus subtilis HU58 and Bacillus coagulans SC208 on gut microbiome dysbiosis using the M-SHIME in vitro model. MegaDuo™ treatment increased microbial diversity, particularly *Bacteroidetes* and *Firmicutes*, which are SCFA producers. In dysbiotic conditions, the probiotic formulation significantly reduced inflammatory markers, like TNF-α (from 9172.6 pg/mL to 6604.7 pg/mL, *p* = 0.0067) and IL-6, while slightly increasing the anti-inflammatory cytokine IL-10. There is no doubt that for this group of drugs, artificial GIT models are the most suitable test systems at the preclinical research stage.

GIT model systems also allow the study of interactions between various drugs and the microbiota, which is becoming increasingly important for understanding their safety. For example, many antibacterial agents can not only affect pathogen populations but also disrupt beneficial microbial communities, leading to dysbiosis and impaired gut health. By using GIT models, researchers can thoroughly investigate these interactions, providing valuable data for the development of safer, more targeted drugs [5].

This suggests that artificial GIT models are also useful test systems for the development of veterinary drugs. For example, study [53] evaluated the impact of *Bifidobacterium thermophilum* RBL67 and prebiotics (FOS, GOS, MOS) on *Salmonella* Typhimurium N-15 colonization in the pig colon. FOS and GOS alone significantly reduced *Salmonella* levels below detection (<4.1 log10 CFU/mL) within three days. Combining *B. thermophilum* RBL67 with FOS or GOS further enhanced inhibition, reducing *Salmonella* counts below detection in two days. The total SCFA concentrations increased by ~29% with FOS or GOS, especially acetate (+38%) and propionate (+28%). The *B. thermophilum* RBL67-FOS combination also boosted butyrate levels by 18% compared to controls. In another study, the effectiveness of bacteriocins (MccJ25) against *Salmonella* was examined under conditions mimicking the pig intestine [56]. MccJ25 remained active for 24 h in simulated swine colonic conditions, with concentrations verified through LC-MS analysis. It demonstrated a consistent inhibitory effect on *Salmonella Newport*, reducing bacterial load by 10-fold beyond natural washout rates. In contrast, reuterin and rifampicin showed minimal or transient effects. The in vitro model has also been used to research the bioavailability of MccJ25, and it maintained stability and antimicrobial activity due to its unique lasso structure, which resists degradation by proteases and extreme pH, making it suitable for gastrointestinal environments. In both research projects, the PolyFermS model was used, which is the most suitable for veterinary applications among the known systems. This test system allowed for the accurate simulation of pig colon conditions, enabling researchers to achieve their study objectives.

It is clear that for use in veterinary drug development, the widely known GIT models require refinement and adaptation. This applies primarily to systems, like SHIME and SIMGI, which are used in pharmacokinetic studies of human drugs [5]. These models can allow researchers to monitor drug dissolution, absorption rates, and interactions with the intestinal mucosa and microbiota in real time. Modified bioreactor systems will be particularly useful for testing the bioavailability and therapeutic effectiveness of veterinary antibiotic, anthelmintic, and antiparasitic agents. These systems will provide critically important information about drug transformations in conditions that mimic the intestines, offering data that would otherwise require extensive animal testing.

### 3.3. Toxicology and Risk Assessment

In the field of toxicology and risk assessment, artificial GIT models have been successfully used to study the effects of potentially hazardous substances on the digestive system and general health of both humans and animals. The most widely used toxicological studies are conducted using the SHIME system to study the safety of products intended for humans. These studies are mainly devoted to studying the effects of pollutants, such as heavy metals and pesticides.

Thus, the results of the cadmium toxic property research based on the SHIME installation are known, which showed their reduction under the influence of probiotic strains of lactobacilli [87]. Some of the following examples are in models mimicking human GIT conditions; however, taking into consideration the importance of toxic substances, the cited work can be illustrative material for veterinary practice as well. Using flame atomic absorption spectroscopy (FAAS), Cd biosorption ranged from 1.0832 ± 0.012 to 3.562 ± 0.03 mg Cd/g of bacterial cells from a 10 mg/L CdCl_2_ solution. *Lactiplantibacillus plantarum* HD 48 exhibited the highest biosorption (3.562 ± 0.03 mg Cd/g). The Cd–lactobacilli complex showed strong stability, with minimal metal desorption after washing (*p* < 0.05). In an in vitro digestion model, Cd bioavailability was reduced by 24.71–41.62%. Another series of studies have focused on arsenic (As) bioavailability and metabolism and its toxicology risk assessment utilizing in vitro models and approaches [88,89,90,91]. The study of Alava et al. [89] assessed the influence of diet’s formula on microbial bioavailability and metabolism. An Asian-type (fiber-rich) diet resulted in higher As bioavailability (81.2%) compared to a Western-type (fat/protein-rich) diet (63.4%). However, Western diet conditions led to increased formation of toxic As species—monomethyl arsonite (MMAIII) and monomethylmonothio arsonate (MMMTA)—after 48 h of colonic digestion. These species had higher permeability across Caco-2 cells (60.5% and 50.5%) than arsenate (AsV, 46.5%) and dimethylarsinate (DMAV, 28%). Yin et al. [91] utilized the SHIME model and examined As metabolism in adults and children exposed to contaminated soils. Arsenic bioavailability in the colon increased 1.4–6.8 times for adults and 1.2–8.7 times for children. Adults had higher As methylation rates (2 µg methylarsenicals/hr/g biomass), three times greater than children. However, children exhibited significantly higher arsenite [As(III)] concentrations (1.5–391.3 µg/L), 2–18 times higher than adults, posing greater health risks. 16S rRNA sequencing identified 20 gut bacterial genera involved in As metabolism.

There are also known examples of studying the toxicity of pesticides in the SHIME system, such as chlorpyrifos, which showed its negative impact on the intestinal microbiota and physiological parameters of the GIT [92,93,94]. At the same time, it showed a destructive effect on Caco-2/TC7 cells that imitate intestinal epithelium, which can lead to damage to the intestinal mucosal barrier and the development of inflammation [95,96]. In the veterinary field, artificial GIT systems are still less commonly used for toxicological studies and risk assessment. Most research in this area is devoted to feed safety, where they are used to assess the impact of contaminants on the GIT, especially mycotoxins, and ways to combat them [97].

Thus, the study of Solís-Cruz et al. [98] evaluated the adsorption capacities of chitosan (CHI) and three cellulosic polymers—hydroxypropyl methylcellulose (HPMC), carboxymethyl cellulose (CMC), and microcrystalline cellulose (MCC)—on six mycotoxins (AFB1, FUB1, OTA, T-2, DON, ZEA) using an in vitro digestive model simulating the poultry GIT. HPMC, CMC, and MCC showed superior adsorption compared to CHI. MCC was the best adsorbent for ZEA (89.7%), followed closely by CMC (83.5%). CHI exhibited moderate binding across five mycotoxins but was ineffective against DON. Cellulosic polymers rely on hydrogen bonding and electrostatic interactions, while CHI performance may improve with molecular modifications, like cross-linking. In this case, a poultry GIT model was used, which proved well and allowed us to fulfill all the tasks set. The same GIT model was used to evaluate the effectiveness of three biosorbents against the poultry diet contaminated with aflatoxin B1 [99]. As a result, aloe powder showed the highest efficacy level against this toxin. Additionally, other authors have studied the effectiveness of some commercial products against two mycotoxins (deoxynivalenol and zearalenone) on the artificial GIT models of pigs and poultry and found that preparations for the enzymatic degradation of mycotoxins are more preferable than adsorbents [65]. In the study of Kolawole et al. [100], the researchers investigated the application of a developed artificial gut model in which they compared 10 mycotoxin binders, revealing that a modified yeast cell wall extract showed the highest efficacy level (over 50%).

All artificial GIT models used in the above studies have successfully evaluated the efficacy of various anti-mycotoxin formulations for use in poultry and livestock. These systems, even the simplest ones based on static principles, can allow researchers to simulate the digestive processes of the animal gut and assess the impact of feed toxins on microbial populations and fermentation efficiency. These studies will be important for determining the toxicity thresholds of various contaminants and their potential to disrupt nutrient absorption and microbial metabolism. At the same time, dynamic models can provide insight into the barrier function of the gut, providing data on how toxins affect permeability and translocation of harmful substances into the bloodstream.

## 4. Technical and Methodological Considerations

### 4.1. Model Design

The development and implementation of artificial gastrointestinal systems studied in various research reflects a wide range of technical innovations and methodological advances aimed at modeling the digestive environment of various animals (Table 1).

The first example is the RUSITEC (Rumen Simulation Technique) developed in the 1970s, which was developed to recreate the condition of the scar in ruminants [39]. This system maintains anaerobic conditions at a constant temperature of 39 °C and slowly moves the inner tank up and down with an electric motor (six times per minute), which perfectly mimics fermentation in the rumen. The model uses fermenters, to which a scar fluid obtained from cows with fistulas is added, which mimics the activity of bacteria over a long period of time. When assessing the digestibility of feed, the Ankom DaisyII static system is a simpler but, at the same time, highly effective alternative for assessing the digestibility of dry matter (DMD) and neutral detergent fiber digestibility (NDFD) in ruminants [101]. The system uses rotating cans with polyester bags in which samples and an enzyme-based scar solution are placed. The DaisyII system is characterized by its ease of use and the ability to process several samples at the same time, which makes it an effective tool for the analysis of powerful nutrients. Despite the movement (rotation of the pistons), there is a lack of dynamic control over digestive parameters, such as pH or the secretion of enzymes characteristic of true dynamic systems. DaisyII has also been adapted for research on non-ruminant animals [102,103,109].

The development of porcine gastrointestinal models is carried out in a dynamic system that mimics the digestive process in the stomach, paying special attention to variables, such as pH, pepsin activity, and gastric emptying [104]. The model uses a three-layer beaker and computer-controlled pumps to regulate the flow of gastric juice. An important feature is the ability to dynamically adjust the pH (from 5.18 to 2.5 for 300 min) and support stirring to simulate peristaltic movements [104]. The TIM-based dynamic model of gastrointestinal digestion in dogs offers a different approach aimed at modeling the digestive and absorption processes of protein and calcium in dogs [76]. This system consists of four compartments, each of which monitors the temperature and pH, imitating the functioning of the stomach and intestines of a dog. Adapting this model to different digestion times depending on the size of the dog allows researchers to study the effectiveness of digestion and the rate of absorption of nutrients depending on the breed. The dynamic nature of this model more accurately reflects the digestive process in dogs than static models, which makes it especially valuable to test the nutrition of pets.

In studies on pigs, the PolyFermS system is a two-stage continuous fermentation system that mimics the proximal part of the colon of a pig [105]. This model uses immobilized fecal microbiota in gel pellets to obtain a homogeneous seed for several enzyme reactors. PolyFermS allows testing of various feed mixtures in parallel by monitoring the stability of microorganisms and the production of short-chain fatty acids, including acetate, propionate, and butyrate. The DIDGI system is a dynamic gastrointestinal system with two chambers designed to mimic digestion in the stomach and small intestine [107]. The DIDGI system with pH, redox, and temperature sensors monitors the passage of the chimus between the chambers using peristaltic pumps and Teflon membranes that simulate the filtering effect in the pyloric region of the stomach. Its ability to control pH changes (starting at 6.5 and reaching 3.10 after 210 min), and reproducing the gastric emptying regime ensures physiological reliability.

In parallel, SalmoSim, a three-part in vitro simulator, reproduces the gastrointestinal tract of Atlantic salmon, including the stomach, pyloric caeca, and middle intestine, while maintaining the microbial communities [108]. The bioreactor system works under certain conditions: the temperature in the stomach area is maintained at 12 °C and the pH at 4.0, while in the pyloric caeca and in the middle intestine, a pH of 7.0 and 7.6, respectively, is maintained. The technical accuracy of SalmoSim allows continuous monitoring of microbial diversity and fermentation, as well as sequencing of 16S rRNA to evaluate the dynamics of the development of microorganisms. In addition, its ability to control the production of volatile fatty acids (VFA) and the level of ammonia makes it possible to control enzymatic processes.

### 4.2. Validation of Models

These models have common features that make them effective tools for veterinary digestive examinations. Dynamic systems, such as SalmoSim, RUSITEC, DIDGI, etc., model digestive processes in real time by continuously regulating pH, enzyme activity, and transit time, accurately reflecting the conditions in vivo. In contrast, static models, such as the DaisyII incubator, provide simpler but carefully controlled conditions for assessing the digestibility of nutrients.

In the RUSITEC model, microbial communities remain stable, and quantitative PCR is used to monitor changes in bacterial populations, especially when problems with pathogens, such as *Clostridium perfringens*, occur. Thanks to the 91% correspondence of bacterial communities in vitro with communities in the scar fluid (including all active taxonomic units with a relative number of higher than 0.8%), RUSITEC provides a reliable and reproducible environment for long-term studies in the scar [39]. In the research, the DaisyII incubator has been used for in vitro long-term scar digestion (240 h) of uNDF [110]. A dynamic system simulating digestion in the stomach of a porcine accurately simulates the digestion of a pig in vivo, achieving a 97% correlation (*p* = 0.0001) with the data on the digestibility of the protein in a living organism, proving its reliability to study the digestion of nutrients under controlled conditions [104]. In dog studies with the TIM model, transit time was a critical factor: simulated gastric emptying took 1 to 3 h and bowel emptying up to 5 h. To measure the degree of protein hydrolysis, HPLC was used, and the bioavailability of calcium was evaluated by atomic absorption spectrophotometry. A TIM-based study showed that nutrient uptake is significantly influenced by transit time: a shorter time promotes faster protein hydrolysis, and a longer transit time improves the bioavailability of calcium [76]. The high reproducibility of the PolyFerms model and the ability to keep microbial ecosystems stable for long experiments (54 days) make it particularly effective for nutritional research and microbial studies in swine [105]. The strength of the DIDGI system is a reliable monitoring of protein degradation kinetics, especially of casein and ß-lactoglobulin, with a correlation coefficient of 0.987 (*p* < 0.001) compared to in vivo results [107]. The field of application of DIDGI is being expanded to study other food matrices, which makes it a universal model for gastrointestinal research. The high microbiological replication of the SalmoSim system (97–98% similarity with in vivo salmon samples) underlines its effectiveness as a tool for dietary research to obtain detailed insight into the effects of different types of feed on the intestinal health of salmon [108].

The use of molecular tools, such as quantitative PCR and 16S rRNA sequencing, expands the ability of these models to study the dynamics of microorganisms, and the monitoring of metabolites by HPLC or gas chromatography makes it possible to obtain detailed information about fermentation processes. Regardless of their differences, all systems try to replicate physiological conditions as accurately as possible, providing researchers with universal platforms.

## 5. Future Perspectives and Advancements

### 5.1. Integration with ‘Omics’ Techniques

The combination of omics techniques, such as genomics, proteomics, microbiomics, and metabolomics, with the artificial gastrointestinal system allows researchers to conduct more comprehensive investigation of the digestive process at the molecular level. Such approaches are crucial for developing detailed models of microbial function and their metabolic outcomes. Many studies have been used as the tools of ‘omics’ techniques [36,43,44,70,76,77,78,80], but some research teams did not explicitly leverage such approaches [8,51,104,107].

A foundational aspect of metagenomics is 16S rRNA gene sequencing, which has been used in numerous studies. This tool provides precise analysis of how different microbial communities (microbiota) are sustained within certain artificial systems. The research results [38] based on 16S rRNA sequencing shows that semi-continuous RUSITEC systems maintain microbial diversity better than batch-style ones (Ankom DaisyII). Expanding this analysis with metatranscriptomics or proteomics could reveal how specific microbes contribute to rumen function in these artificial systems. Other tools, like ITS sequencing, have been applied to determine the mycobiome in three artificial rumen systems. This technique defined the fungal population within the Ankom DaisyII vessels, displaying a sharp and continuing shift in alpha diversity for the whole term of the trial, while the mycobiome in the RUSITEC systems retained a more constant alpha diversity and uniformity. The 16S rRNA sequencing and QIIME (Quantitative Insights into Microbial Ecology) platform tools leveraged in another study [45] revealed differences in microbial diversity depending on the type of inoculum (cecal or fecal) and its status (fresh or frozen) within chicken research. This information can be valuable for the selection of sample sources and preservation methods for subsequent trials. By integrating sequencing data with metabolic analysis, research sheds light on microbial metabolic outputs, such as SCFAs and branched-chain fatty acids (BCFAs), linking microbiota dynamics to functional outputs. The CALIMERO-2 model employs 16S rRNA gene sequencing [46] with further analysis by teh QIIME2 bioinformatics platform and metabolite quantification techniques. Microbiota profiling via the 16S rRNA sequencing enables researchers to determine the relative abundance of major bacterial phyla, including *Bacteroidetes*, *Firmicutes*, and *Proteobacteria*, which are predominant in cecal samples. It also demonstrates that bacterial alpha diversity decreases after inoculation but increases over 72 h, becoming more similar to the original inoculum. This indicates the system’s capacity to maintain the microbiota structure over time. The production of acetate, propionate, and butyrate over time was monitored, confirming that microbial metabolic function was maintained. Differences in metabolite ratios (e.g., lower butyrate vs. propionate) revealed that diet and chicken breed may influence SCFA profiles, which can be modeled in vitro for further testing. This consolidation of ‘omics’ approaches offers a close-up picture of how dietary interventions or environmental conditions may change microbial compositions, which is crucial for understanding gut health and metabolism in chickens.

Studies employing the PolyFermS model widely use integration with ‘omics’ techniques. 16S rRNA sequencing allows a deep understanding of microbial diversity and phylogenetic composition, revealing dominant phyla, like Firmicutes, Bacteroidetes, and Proteobacteria, in both in vitro and in vivo samples [62,105,106]. The data from qPCR tools have confirmed the results of 16S rRNA gene sequencing, offering a complementary approach to analyze microbiota diversity and abundance [105]. Research [62] has used the metagenomics imputation method Phylogenetic Investigation of the Communities by Reconstruction of Unobserved States (PICRUSt2) to predict the functional pathways from the observed microbial profiles. Two studies that used the PolyFermS system conducted a metabolomics investigation through HPLC to analyze SCFAs, which are key indicators of gut health and microbial activity. In the first study, this approach helped to reveal that glycerol supplementation led to a reproducible increase in butyrate production in all models, while reuterin manufacture was linked to the inhibition of harmful bacteria, such as Enterobacteriaceae [106]. In the second one, the metabolite data helped the researchers confirm that metabolic activity remained stable over the long-term experiment; notably, butyrate production increased over time [105]. This conceptual synthesis of metagenomics, qPCR, and metabolomic profiling allowed us to track changes in microbiota composition and function in real time, providing a detailed understanding of the microbial ecosystem’s response to the interventions. In the research utilizing the SalmoSim model, ‘omics’ approaches employing NGS and 16S rRNA were integrated effectively [108]. In this study, high-throughput sequencing tools, like Illumina HiSeq, were employed to analyze microbial DNA extracted from samples collected from both the in vitro SalmoSim system and live salmon. Metagenomic analyses allow the ability to digest plant-based feeds or produce in-chain fatty acids. 16S rRNA gene sequencing was employed in study [39] related to the RUSITEC system to identify the operational taxonomic units (OTUs) across 17 bacterial phyla, with Bacteroidetes and Firmicutes being dominant. Metabolomics was conducted using direct injection mass spectrometry with reverse-phase LC-MS/MS.

Study [8] did not directly employ “omics” technologies, such as genomics or proteomics, but integrated quantitative nutrient data to establish predictive models for rumen fermentation outcomes, including methane and acetic acid production. By analyzing the variables (neutral detergent fiber, acid detergent fiber, etc.), the study sought to simulate the complex biochemical processes within the rumen environment, analogous to how ‘omics’ techniques identify correlations between vast molecular datasets and biological outcomes. In the study of Ménard et al. [107], the integration of the DIDGI system with “omics” technologies is implicit in its focus on molecular-level food digestion processes but the system facilitates detailed proteomic studies by analyzing the kinetics of proteolysis in food matrices. The DIDGI system offers a controlled environment where ‘omics’ approaches can be applied to identify the transformation and bioavailability of nutrients, complementing ‘omics’ studies focusing on digestive biochemistry. Study [104] on dynamic gastric digestion systems in pigs does not explicitly discuss the integration of ‘omics’ techniques. Since the study focused on protein digestion then proteomics, it could offer in-depth insights into how various diets affect the protein breakdown process in the stomach.

### 5.2. Advancements in Bioengineering

Semi-continuous design of RUSITEC PP and RUSITEC Prime allows for the maintenance of stable microbiota and VFAs over extended periods (up to 120 h). In contrast, the batch system (Ankom DaisyII) lacks this feature, leading to a decline in pH and microbial diversity over time [38]. The key bioengineering innovation of the research [8] lies in constructing a predictive framework using machine learning to model how dietary compositions (total mixed rations, or TMRs) affect methane and acetic acid production without the need for invasive in vivo experimentation. Traditionally, such analyses require rumen-cannulated animals for continuous sample collection, which is labor-intensive, expensive, and ethically challenging. The major innovation of the CALIMERO-2 model [46] is the creation of a four-unit system with semi-permeable membranes and dialysis capabilities that can continuously remove microbial metabolites by collecting real-time data on microbial fermentation without causing product inhibition, preventing metabolome accumulation and thus better maintaining microbiota activity over long experimental periods.

In future versions of the DIDGI model, membranes will be added to mimic nutrient absorption, enhancing the physiological relevance of the system [107]. These features enable researchers to study not only how food breaks down but also how nutrients are absorbed and utilized by the organism. Software of the dynamic system simulating pig gastric digestion incorporates data from in vivo studies to predict digestion parameters, such as pH, enzyme secretion rates, and gastric emptying rates. These predictive models enable the system to adjust in real-time based on the simulated animal’s physiology, improving the accuracy and relevance of the results obtained [104]. PolyFermS allowed the simultaneous comparison of different experimental treatments using parallel second-stage reactors inoculated with effluent from the primary reactor. This innovation enables efficient testing of various conditions, such as dietary additives or antibiotics, without host interference [62,105,106]. The modularity of the TIM-based [76] model means it can be adapted to simulate the GI tract of other animals or to include additional factors, such as bile secretion, microbial interaction, or even immune response, making it a versatile platform in the field of bioengineering for veterinary and nutritional research.

### 5.3. Expansion to Novel Species and Need for Models Mimicking Poultry, Aquaculture, Pets, etc.

The ability to study digestion in a controlled, ethical, and non-invasive way opens doors for research into wild or endangered species where in vivo studies are not feasible. The semi-continuous systems, particularly RUSITEC, offer a viable platform to model the digestive processes of non-ruminant herbivores, such as horses or rabbits [38]. These animals also rely on microbial fermentation in their hindgut or large intestine, which could be simulated by adjusting the inoculum and fermentation conditions. Although the CALIMERO-2 model [46] is specifically designed for chicken ceca, the system holds potential for expanding research to other species with similar gut microbiota environments consisting of such bacterial groups as Bacteroidetes, Firmicutes, and Proteobacteria. The Ankom DaisyII incubator, initially developed for ruminants, has been adapted successfully for horses, donkeys, camelids, rabbits, and Guinea pigs [101]. While previous in vitro fermentation models utilized PolyFermS platform primarily targeted human and child microbiota, these studies [62,105,106] successfully adapted the model for chicken cecal and swine fecal microbiota. The study proposes the potential for expanding the SalmoSim system to simulate the GIT of other fish species [108]. Atlantic salmon was chosen for the initial development due to its significance in aquaculture; however, the principles and methodology described here are applicable to other teleost species. Different fish species exhibit diverse gut morphologies, diets, and microbial communities, which makes SalmoSim a promising model for adaptation.

### 5.4. What Needs to Be Done or Cannot Be Done by Using aGIT Models

Even if it has been applied on several occasions, aGITs are still considered as simplified representations of real animal or human digestive systems. These systems often simplify complex in vivo conditions, which may lead to inaccuracies in replicating the full spectrum of gastrointestinal processes, but this is why they are called “models”. Moreover, species-specific variability is an additional task that deserve better attention from the research teams. These models may not fully account for the unique physiological differences across various animal species; on most occasions, they are very specific to narrow groups of animals. Almost all systems are associated with limited microbial diversity, where the microbial communities in aGIT models may not accurately represent the diversity and dynamics of gut microbiota found in live animals/humans.

Cost and maintenance are still an issue that limits their applications. Dynamic aGIT models, such as TIM, can be expensive to develop, maintain, and operate. They need qualified technical expertise. Using and interpreting results from aGIT models require specialized knowledge, which may limit accessibility for some researchers or educators. There are the issues with standardization of protocols and the integration of large volumes of data while utilizing aGIT systems.

Topics that cannot be solved by GIT modeling or those not yet solved are related to behavioral and neurological interactions. Simply, the aGIT models cannot replicate the gut–brain axis or the behavioral effects of gastrointestinal health. Moreover, immune system dynamics is a subject that will be very difficult to model. The interaction between the gut and the immune system is complex and cannot be fully simulated by GIT models. Or even long-term effects and chronic conditions or dietary interventions are difficult to study using short-term GIT simulations.

Models represent general conditions; however, with real consequences, environmental influences can be important factors as well. Factors affecting gut health, like stress, climate, and external environmental conditions, are beyond the scope of aGIT models or individual variability is a factor, as aGIT systems cannot account for genetic, epigenetic, or lifestyle differences among individual animals.

## 6. Conclusions

Artificial gastrointestinal tract models have become valuable tools in veterinary research. Their application has significantly advanced our understanding of feed digestibility, microbial interactions, disease mechanisms, and the pharmacokinetics of veterinary drugs. Importantly, these models support the development and testing of probiotics, prebiotics, antimicrobial agents, and therapeutic compounds, including antibiotics and antiparasitics. Emerging research highlights the potential of integrating aGIT systems with high-throughput ‘omics’ technologies—such as genomics, proteomics, and metabolomics—to capture a more comprehensive molecular portrait of host–microbiota–diet interactions. This integrative approach is expected to drive innovations in precision nutrition, drug efficacy testing, and toxicological assessments in veterinary medicine, solidifying the role of artificial aGIT models as responsible platforms for future translational research.

## Figures and Tables

**Table 1:** Overview of the artificial GUT models employed in veterinary practice.

Model Name	1st Release	Type of Model	Core-Animals	Controlled Parameters	Sections of GI	Primary Applications	Reference
RUSITEC	1970s	Dynamic	Cattle, Sheep	Temperature, anaerobic conditions, nutrient supply	Rumen	Microbial dynamics, metabolome studies	[39]
Ankom DaisyII	1994	Static	Ruminants, non-ruminants	Temperature, digestion duration	Rumen, stomach	Digestibility studies	[101,102,103]
TIM-based	1999	Dynamic	Dogs	pH, temperature, enzyme secretion, gastric emptying	Stomach, small intestine	Drug release, nutrient bioavailability	[76]
Dynamic System Simulating Pig Gastric Digestion	2008	Dynamic	Pigs	pH, gastric fluid secretion, pepsin activity, digesta volume, mixing	Stomach	Protein digestibility in pigs; simulating gastric digestion	[104]
PolyFermS	2012	Dynamic	Pigs, chickens	pH, temperature, anaerobic conditions, flow rates	Stomach, small intestine, colon	Microbial fermentation, nutrient digestion	[61,105,106]
DIDGI	2014	Dynamic	Pigs	pH, temperature, enzyme secretion, chyme flow	Stomach, small intestine	Digestion kinetics, protein hydrolysis studies	[107]
SalmoSim	2020	Dynamic	Atlantic Salmon	pH, temperature, feeding cycles	Stomach, pyloric caeca, midgut	Studying microbial activity, nutrient digestion in fish	[108]
CALIMERO-2	2021	Dynamic	Chickens	pH, temperature, transit times, and enzymatic activities	Stomach and intestines (ceca)	Nutrient absorption, feed additives	[46]
MPigut-IVM	2021	Dynamic	Piglets	pH, temperature, redox potential, transit times, stirring speed	Colon	Impact of dietary changes and pathogenic bacteria on microbiota	[67]

## Data Availability

No new data were created or analyzed in this study.

## Abbreviations

The following abbreviations are used in this manuscript:

16S rRNA gene sequencing	Amplicon-based sequencing method that is used to identify and classify bacteria present in bulk and complex biological samples
aGIT	Artificial gastrointestinal tract
DOAJ	Directory of open access journals
GIT	Gastrointestinal tract
HPLC	High-performance liquid chromatography
LD	Linear dichroism
MDPI	Multidisciplinary Digital Publishing Institute
NGS	Next-generation sequencing
Omics	High-performance analytical methods used to study the various types of molecules that make up a living organism, including DNA, RNA, proteins, and metabolites
qPCR	Quantitative PCR
SCFAs	Short-chain fatty acids
TLA	Three-letter acronym
uNDF	Undigested NDF
